# Factors Associated with Objectively Measured Physical Activity in Patients with Seropositive Rheumatoid Arthritis

**DOI:** 10.3390/ijerph17239008

**Published:** 2020-12-03

**Authors:** Sandra Haider, Michael Sedlak, Ali Kapan, Igor Grabovac, Thomas Lamprecht, Ludwig Erlacher, Michael Quittan, Karl Heinrich Fenzl, Thomas Ernst Dorner

**Affiliations:** 1 Department of Social and Preventive Medicine, Center for Public Health, Medical University of Vienna, 1090 Vienna, Austria; sandra.a.haider@meduniwien.ac.at (S.H.); n01542415@students.meduniwien.ac.at (M.S.); ali.kapan@meduniwien.ac.at (A.K.); thomas.dorner@meduniwien.ac.at (T.E.D.); 2 Karl Landsteiner Institute for Autoimmune Disease and Rheumatology, 1100 Vienna, Austria; t.lamprecht@sportunion.at (T.L.); ludwig.erlacher@gesundheitsverbund.at (L.E.); karlhfenzl@gmail.com (K.H.F.); 3 2nd Medical Division, Rheumatology, Klinik Favoriten, 1130 Vienna, Austria; 4 Karl Landsteiner Institute for Remobilization and Functional Health, 1130 Vienna, Austria; mq@rehab-hietzing.at; 5 Karl Landsteiner Institute for Health Promotion Research, 3454 Sitzenberg-Reidling, Austria; 6 Social Insurance Fund for Public Service, Railway and Mining Industries, Health Promotion Facility Sitzenberg-Reidling, 3454 Sitzenberg-Reidling, Austria

**Keywords:** physical activity, accelerometer, rheumatoid arthritis

## Abstract

*Background:* Rheumatoid arthritis (RA) is a chronic autoimmune disease, which is associated with low levels of physical activity (PA). However, the factors related to low physical activity levels have rarely been studied. *Methods:* In this cross-sectional study, 70 seropositive RA patients were included. Physical activity was objectively assessed with an ActiGraph GT3X+ accelerometer. In addition, body mass index, smoking status, work ability, and clinical parameters (functional disabilities, disease activity, disease duration, pain, and inflammation parameters) were measured. *Results:* RA patients performed a mean of 215.2 (SD: 136.6) min a week of moderate physical activity and 9.1 (SD: 26.3) min of vigorous physical activity. The total amount of moderate and vigorous physical activity (MVPA) was associated with BMI, and functional disabilities. In addition, non-smokers and patients with better work ability did more MVPA. No association could be seen with disease activity, disease duration, pain, and inflammatory markers. After mutual adjusting of all the variables, only BMI showed a significant relationship with MVPA. *Conclusions:* RA patients perform de facto no physical activity with vigorous intensity. Factors related to low physical activity are BMI, functional disabilities, workability and smoking status, whereas due to the study design no causal and temporal link could be made.

## 1. Introduction

Rheumatoid arthritis (RA) is a chronic autoimmune disease that usually presents as symmetrical polyarthritis, particularly of the hands, feet, and other synovial joints [1,2]. The prevalence of RA is approximately 1% in the general European population [3,4]. It is associated with pain, stiffness, swelling, fatigue, and sleeping problems [5], having a debilitating effect on functionality. RA is also associated with cachexia [6], low quality of life (QoL) [7,8], limitations in everyday life [9,10], lower work ability [11,12], and sexual problems [13]. 

There is a wealth of evidence supporting the beneficial effects of physical activity (PA) in improving joint health, physical function, and mental well-being, as well as reducing cachexia and fatigue in patients with RA [14,15]. Additionally, it was shown that physical activity is a protective factor in the etiology of RA. As such, there was a statistically 35% lower risk for developing RA among women in the highest category of leisure-time activity as compared to women in the lowest category (less than 20 min per day of walking/bicycling and less than 1 h per week of exercise) [16]. Consequently, the European League Against Rheumatism (EULAR) recommends 150 min of moderate physical activity per week, or 75 min of vigorous physical activity or a combination of both, with a minimum duration of 10 min per session. In addition, it is recommended that resistance training should be performed twice a week [17]. Even though RA patients commonly report that they are aware of the positive effects of physical activity [18,19], systematic reviews have shown that physical activity is lower in RA patients than in healthy controls [14,20]. In a cross-sectional study of 21 countries, only 13.8% of patients with RA reported regular physical activity [21]. 

Associations between clinical factors and the total amount of physical activity have also been reported. A 2017 review reported that higher moderate to vigorous physical activity (MVPA), as measured by a questionnaire, correlated with lower disease activity, the number of comorbidities, the number of hospital admissions, low body mass index, low blood pressure, a lower risk for insulin resistance, and improved physical function [22]. Furthermore, it was also reported that physical activity frequency did not differ significantly between patients with early- and long-term RA, whereas 37% of early and 43% of long-term RA patients did not achieve the MVPA recommended levels [23]. In terms of the effects of physical activity levels on functional limitations, one study reported only a moderate association between physical activity and functional limitations, as measured by the Health Assessment Questionnaire Disability Index (HAQ-DI) [24]. In addition, a study by McKenna et al. [25] demonstrated a significant negative relationship between physical activity and functional limitations, as well as inflammatory parameters.

To sum up, although the literature suggests that physical activity is an essential part of RA therapy, very few people meet the recommendations. Factors affecting physical activity behavior in this population have rarely been studied, especially where physical activity levels are objectively measured, and it would be necessary to produce tailored physical activity recommendations. Hence, the aim of the present study was to assess the association between health-related factors such as body mass index and smoking status with physical activity in seropositive RA patients.

## 2. Material and Methods

This monocentric cross-sectional study, which was conducted from September 2017 to November 2019 in Vienna, Austria, was approved by the local ethics committee (EK 17-039-0417) and complied with the Declaration of Helsinki [26]. Patients were recruited during their regular scheduled visit to the rheumatology outpatients’ clinic. The following inclusion and exclusion criteria were applied: (1) Age 36–80 years (mid-adulthood and late-adulthood) [27]; (2) seropositive RA according to the EULAR guidelines [28]; (3) sufficient knowledge of the German language; and (4) ability to walk at least 20 m without a break. In addition, those who: (1) Did not want to wear the accelerometer; (2) had a planned in-patient stay or holiday within the following week; or (3) had an acute disease (e.g., respiratory infection) were excluded from the study.

### 2.1. Measurements

Physical activity was objectively assessed with an ActiGraph GT3X+ accelerometer (ActiGraph, Pensacola, FL, USA), which was a valid and reliable tool for measuring physical activity in 3 axes [29,30]. Participants were instructed to wear the accelerometer upon arising (except for swimming or other water activities) until going to bed for 7 consecutive days. The accelerometer was worn on the dominant side of the hip [31]. The data measured by the accelerometer were subsequently summarized in time segments of 10 s (epochs). Data analysis was undertaken using ActiLife v6 software (ActiGraph, Pensacola, FL, USA), with the following rules being applied: (1) Periods of more than 60 min without accelerometer activity were defined as “non-wearing time.” During this non-wearing period, episodes of 1 to 2 min of activity (with a count of up to 100) were allowed [32]. (2) A valid day was defined as ≥10 h of wearing time during 24 h [30]. (3) As has been reported in previously published studies, only persons having at least 4 valid days were included in the final analysis [33,34]. If there were fewer than 7 valid days, the time spent in each movement category was divided by the number of valid days. The resulting value was then multiplied by a factor of 7 to obtain a comparable number. The cut-off-values of Troiano et al. [32] were used for all movement, even if this lasted less than 10 min, i.e., time spent in light (100–2019 counts per min), moderate (2020–5998 counts per min), and vigorous (5999–∞ counts per min) physical activity. In addition, MVPA was calculated as time spent in moderate and vigorous physical activity, without multiplying the vigorous physical activity by two.

To assess the body mass index (BMI), body height (in meters) and body weight (in kilograms) were measured without shoes or heavy clothes using a calibrated scale. BMI was categorized as underweight (<18.5 kg/m^2^), normal weight (18.5–24.9 kg/m^2^), overweight (25.0–29.9 kg/m^2^), and obese (≥30 kg/m^2^) [35].

Functional disabilities were assessed with the Health Assessment Questionnaire (HAQ), a validated instrument for patients with RA [36,37]. This questionnaire included 20 items, divided into 8 categories (dressing, arising, eating, walking, reach, grip, hygiene, and other daily activities). The disability index (HAQ-DI) was calculated by taking the mean of the highest value of each category. The HAQ-DI score varied from 0 (lowest score) to 3 points (highest score). A score of 0–1 was categorized as “mild to moderate” disability, 1–2 as “moderate to severe” disability, and >2 as “severe to very severe” disability [38].

Disease activity was measured with the Clinical Disease Activity Index (CDAI), a validated measurement for seropositive RA patients [39], with the following disease activity levels: “remission” (CDAI ≤ 2.8), “low disease activity” (CDAI > 2.8 and ≤10), “moderate disease activity” (CDAI > 10 and ≤22), and “high disease activity” (CDAI > 22).

As laboratory parameters, C-reactive-protein (CRP) and blood sedimentation were measured in an accredited laboratory.

Disease duration (years) was based on the elapsed time from when RA was first diagnosed.

Pain intensity was assessed with the HAQ-VAS-Pain-Scale, asking the question, “How would you rate your pain over the past week on a scale ranging from 1 to 100 (1 = no pain at all; 100 = maximum pain)?” [36,37].

The information about existing comorbidities was based on self-reporting. The Charlson Comorbidity Index (CI) was calculated by summing up the weighted comorbidities and by adding a score for age [40]. Participants were divided into “mild comorbidities” (CI < 2), “moderate comorbidities” (2 ≤ CI ≤ 4), and “severe comorbidities” (CI ≥ 5).

Muscle strength was obtained by measuring the handgrip strength with a JAMAR^®^ handheld dynamometer (JAMAR, Lafayette, LA, USA), with measurements being conducted for both hands 3 times each. Participants were seated with their arms bent at the elbow, and they were instructed to compress the dynamometer with maximum effort [41]. The maximum value was then taken for the calculation. The sex- and age-specific values of the dominant hand of the European population were used to calculate the percentage of patients “under the reference values” [42], as shown in Table 1.

Sex, age, relationship status (in a relationship, without a relationship), education level (compulsory school or no degree, secondary school, higher education), smoking status (yes/no), and employment status (retired, employee, self-employed, unemployed, in education, homemaker) were measured. In addition, work ability was measured using the work ability score (WAS; Work Ability Index Single-Item Scale) by asking “*How would you rate your work ability at present, compared to the best ever work ability you had in your life on a scale from 0 to 10?”* (0 = unable to work; 10 = best work ability) [43].

### 2.2. Statistical Analyses

Depending on the distribution, the results were expressed in the mean (standard deviation, SD) or median (25% and 75% percentiles) for continuous variables and as percentages for categorical variables. The min spent on MVPA in subgroups was calculated using independent t-tests To explore the associations between the clinical factors, sociodemographic and health-related factors (body mass index and smoking status), and the dependent variable (MVPA), linear regression analyses were performed. In these analyses, we included all the variables showing a *p*-value <0.2 [44]. Univariate linear regression analyses (Model 1) were first conducted, using the variables as continuous variables. This was followed by the construction of linear regression models, adjusted for the sociodemographic variables of sex, age, and education (Model 2). The final multiple linear regression model (Model 3) was then constructed, including all the variables, adjusted for each other mutually. Results were expressed as regression coefficients and the 95% confidence level (CI). For all the statistical analyses, IBM^®^SPSS^®^Version 20 software (IBM Corp., Armonk, NY, USA) was used. All the tests were 2-sided, and a *p*-value of <0.05 was considered to be statistically significant.

## 3. Results

Initially, 83 people fulfilled the inclusion criteria, and valid measurements were obtained from 70 patients, of which 72.9% were female. Participants had a mean age of 57.9 (SD: 9.5) years. The youngest participant was 37 years of age, and the oldest was 76 years of age (Table 1). According to the BMI, 22.9% were of normal weight, and 75% were obese or overweight. For the other categories, 40% were smokers, 38.6% were working at the time, and 50.0% were already of pension age. Among the sample, 62.9% had a handgrip strength score under the age- and sex-specific reference values for healthy European adults. The participants had lived with RA for 8 years on average (25–75% percentile: 5–12 years). The current median pain intensity was 30 (10–50) points. The majority of the participants were in “remission” or had “low disease activity,” and three out of four had “mild to moderate” functional disabilities. In addition, a quarter of the population had increased CRP values. Comparable results were found in the blood sedimentation results. The majority of the RA patients had moderate comorbidities. In detail, 14.3% had diabetes mellitus type 2, and 11.4% reported having chronic lung disease. Moreover, 5.7% reported having heart insufficiency, with the same frequency being reported for peripheral vascular diseases, liver disease, and a solid tumor, respectively. Two persons reported diabetes mellitus type 1 or hemiplegia. Additionally, one person had chronic leukemia, and another one an ulcer disease.

When looking at the accelerometer data (Table 2), RA patients did 1222.9 (SD: 484.5) min per week of light physical activity. They had an average of 215.2 (SD: 136.6) min a week of moderate physical activity and 9.1 (SD: 26.3) min a week of vigorous physical activity. Adding up the moderate and vigorous physical activity, RA patients did 224.3 (SD: 146.5) min per week of MVPA, on average. 

Patients of normal weight did significantly more MVPA than those classed as overweight and almost double the physical activity of those classed as obese (Table 3). In addition, patients who did not smoke and patients who were working performed significantly more MVPA (*p* < 0.05). Even though not statistically significant, younger patients, patients with a higher work ability score, patients with lower functional disabilities, patients with lower pain intensity, patients with lower disease activity, and patients with only moderate comorbidities spent markedly more minutes per week performing MVPA than their counterparts. For sex, education level, handgrip strength, laboratory findings for inflammation, and disease duration, the data were inconclusive in this regard. 

The results of the univariate linear regression analyses (Model 1), treating variables continuously showed that HAQ-DI, BMI, smoking status, and the WAS score were significantly associated with MVPA (Table 4). After adjusting for sex, age, and education (Model 2), only BMI was significantly associated with MVPA. After adjusting all the variables for each other (Model 3), only BMI showed a significant relationship with MVPA.

## 4. Discussion

The findings of this study show that RA patients of normal weight, non-smokers, and patients with a high work ability do more MVPA than those with a higher BMI, smokers, and those not in employment. However, after adjusting all the variables for each other, only BMI shows a significant relationship with MVPA. Furthermore, the data demonstrate that vigorous physical activity is rarely undertaken by seropositive RA patients.

The finding that RA patients do little vigorous physical activity is in agreement with the results of a study of 50 RA patients by Hernández-Hernández et al. [45], who came up with a median value of 0.6 (0.2–1.6) min/day. In our study, the corresponding values were 0.2 (25–75% percentile: 0.1–0.5) min/day. When looking at the levels of moderate physical activity, our data are with a median of 25.6 (25–75% percentile: 17.0–40.6) min/day, again comparable to those obtained by Hernandez-Hernandez et al. [45], who reported 22.0 (SD: 15.0) min per day. The amount of MVPA with a media of 26.0 (25–75% percentile: 17.3–40.8) min/day was higher than that reported in a US-based study [46], where it was reported that patients (disease duration: 14 years; aged 55 years) spent a median value of 14 min/day. The results were also higher than the results of an English study (disease duration: 7 years; aged 55 years), which reported a mean value of 18 min/day [47]. However, the results of this study are comparable to the results of Hörnberg et al. [23], who reported a median duration of 34 min/day in early RA and 26 min/day in long-standing RA, and are also comparable to the results of Khoja et al. (median disease duration of 14 years, aged 58 years), who reported a median value of 36 min/day [48]. The variation between studies may partly be explained by the differences in disease severity. Furthermore, comparisons are hampered by the use of different activity monitors, with differences in device sensitivity, sampling, and data filtering, as well as the use of proprietary algorithms and cut-off values for the data handling. With the present data, the reasons for the low amount of vigorous physical activity can only be hypothesized. As proposed by Baslund et al. [49], the fact that many RA patients have concerns about potentially increasing pain and exacerbated disease activity may lead to them avoiding high levels of vigorous physical activity. As vigorous physical activity is also recommended for RA patients [17], and with regard to our results, healthcare professionals should point out that vigorous physical activity is also health-enhancing, and they should help patients to dispel some of the fear that vigorous physical activity can increase disease activity.

The amount of physical activity was similar to the measured physical activity in patients with type 2 diabetes (disease duration: 13 years; aged 65 years) [50]. In this study Mathe and colleagues reported 22.2 (SD: 19.4) min/day of moderate and 0.2 (SD: 0.71) min/day of vigorous physical activity. Our results are also comparable to women with a low bone mineral content (mean age 64.5 years), who conducted 25.7 (SD: 22.6) min of MVPA/day [51]. However, men included in this study did 41.3 (SD: 25.3) min of MVPA/day more physical activity. When putting our results in relation to people with knee osteoarthritis and overweight (*n* = 160; aged 66 years), who came up to 10.6 (SD: 8.9) min of MVPA/day, we can see that our included RA patients did more PA [52].

When looking at the factors associated with MVPA, BMI shows the only association when adjusted for other factors. This means, when adjusted for all other variables, one minute more of MVPA per week, was associated with a 0.35 lower BMI. As in another study by Albrecht and colleagues [53], the percentage of obese patients in the RA population was higher than in the general population. In addition, the fact that overweight or obese RA patients do less physical activity than patients of normal weight has also been reported in a study by Hugo et al. [54]. As disease activity has been shown to be higher and disabilities more severe in obese RA patients, increasing physical activity in these patients might be of particular importance [55], especially when considering that physical activity can reduce the inflammatory process, metabolic syndromes, and other comorbidities (osteoarthritis, depression, diabetes) [54,55].

We also found that non-smokers are more likely to do more physical activity. That smoking is an established risk factor for developing seropositive RA has already been shown [56]. In this context, it has to be mentioned that the percentage of smokers in the present sample was high (40%), especially when compared to the general Austrian population (22% in women and 26% in men) [57], and also to other RA patients [56]. This could be attributed to the relatively high number of participants with only primary education.

It was also found that patients in employment do more physical activity. This might be due to the fact that retired people are older and have a higher disease activity, as shown by our data. However, the finding contradicts the study of Qvarfordt et al. [58], who reported that RA patients who were working and those on sick leave failed to fulfill the recommendations, whereas retired patients were more likely to meet the recommendations. The authors hypothesized that retired RA patients had more free time for physical activity than those who were working. 

Clinical parameters such as disease activity, pain intensity, more severe comorbidities, lower work ability, and more functional disabilities were not found to be significantly associated with MVPA; however, the estimates for MVPA are clearly (up to two times) higher in patients with a lower clinical disease burden. An association between functional disability, as measured with the HAQ-DI, and MVPA was found by Prioreschi et al. [59], who reported that the HAQ-DI score was negatively correlated with physical activity (r = −0.343, *p* = 0.026). Khoja et al. [48] also found an association with very light (r = −0.277), light (r = −0.261), and moderate physical activity (r= −0.384). 

A possible limitation of this study is the cross-sectional design, making a causal and temporal link impossible. Although the sample size in our study was high in comparison to other accelerometer studies in RA patients [33,45,59], the sample size was not high enough to reach a satisfactory statistical power for many of the analyses. A more appropriate study design would be the multicenter design using the same methods that would allow for a more satisfactory sample size, which should be considered for future studies. Furthermore, stationary sports, such as the use of a cycle trainer and weight training, in addition to water sports, were not recorded, which could lead to an underestimation of the total physical activity. It has also been considered that the included participants wore the accelerometer for 7 days, whereas at least 4 days have to be valid. This method is based on previously published literature [33,34]. However, the extrapolation of the data might influence the results.

The fact that we undertook the recruitment in a rheumatology outpatients’ clinic might also bias the results, as patients with lower disease activity and overall better health status were more likely to be included. A strength of this study is that the data were objectively measured, providing objectively measured data with high validity and reliability [60,61].

## 5. Conclusions

In conclusion, we found that vigorous physical activity was, de facto, not performed by RA patients. Healthcare professionals should point out that vigorous physical activity is also health-enhancing for RA patients. Additionally, RA patients of normal weight, non-smokers, with lower functional disabilities, and those currently employed achieved more MVPA, whereas due to the study design, no causal and temporal link could be made. No significant association could be seen with disease activity, disease duration, pain, and inflammatory markers. Consequently, the need for regular physical activity needs to be emphasized, especially in overweight and obese patients. 

## Figures and Tables

**Table 1:** Characteristics of the study sample.

Characteristics	*n* = 70
Female, %	72.9%
Age (years); m (SD)	57.9 (9.5)
In a relationship, %	72.9%
Education level	
Primary school	52.9%
Secondary school	37.1%
Higher education	10.0%
Body mass index (kg/m^2^), %	
Normal weight: 18.5–24.9 kg/m^2^	22.9%
Overweight: 25.0–29.9 kg/m^2^	34.3%
Obese: ≥30.0 kg/m^2^	42.9%
Smoking, yes, %	40.0%
Working at the moment, yes, %	38.6%
Work ability, WAS score; m (SD)	6.6 (1.9)
Handgrip strength	
Absolute, kg; m (SD)	28.3 (12.0)
Under the reference values ^1^	62.9%
Relative, kg/kg; m (SD)	0.36 (0.14)
Functional disabilities; HAQ-DI	
Mild to moderate; %	76.8%
Moderate to severe; %	20.3%
Severe to very severe; %	2.9%
Disease activity; CDAI	
Remission; %	38.6%
Low; %	37.1%
Moderate; %	8.6%
High; %	2.9%
CRP, mg/l; median (25–75% percentile)	2.0 (1.1–5.0)
Over the reference values ^2^	24.3%
Blood sedimentation, mm; median (25–75% percentile)	24.5 (13.3–31.0)
Over the reference values ^3^	14.7%
Disease duration; years; median (25–75% percentile)	8 (5–12)
Pain last week; points (25–75% percentile)	30 (10–50)
Comorbidities	
Moderate comorbidities	70.0%
Severe comorbidities	30.0%

Normally distributed data are shown in mean (m) and standard deviation (SD); not normally distributed data are shown in median with 25% and 75% percentiles. WAS = Work Ability Index Single-Item Scale; HAQ-DI = Health Assessment Questionnaire Disability Index; CDAI = Clinical Disease Activity Index. Differences between the groups in categorical variables were calculated using chi-squared tests or f-tests if ≤5 were in the group. For the metric data, independent t-tests or u-tests were used. ^1^ Sex- and age-specific values of the dominant hand of the European population were used [42]. ^2^ According to the reference range (0–5 mg/L) of the laboratory. ^3^ According to the reference range (0–40 mm) of the laboratory.

**Table 2:** Amount of physical activity.

Amount of Physical Activity	m (SD)
Light intensity; min per week; m (SD)	1222.9 (484.5)
Moderate intensity; min per week; m (SD)	215.2 (136.6)
Vigorous intensity; min per week; m (SD)	9.1 (26.3)
Moderate and vigorous intensity; min per week; m (SD)	224.3 (146.5)

Data are shown in mean (m) and standard deviation (SD).

**Table 3:** Comparison of the mean values for time (min) spent in moderate and vigorous physical activity (MVPA) in subgroups.

		MVPA Per Week;	*p*
		Mean (SD)	
Sex	Male (*n* = 19)	243.1 (144.2)	0.761
	Female (*n* = 51)	229.8 (167.2)	
Age	≤59 years (*n* = 38)	261.7 (199.9)	0.109
	≥60 years (*n* = 32)	199.9 (149.5)	
Education level	Compulsory school or no degree (*n* = 37)	215.7 (156.3)	0.553
	Secondary education (*n* = 26)	251.2 (164.9)	
	Tertiary education (*n* = 7)	261.1 (177.4)	
Body mass index ^1^	Normal weight (*n* = 16)	326.1 (217.5)	0.008
	Overweight (*n* = 24)	244.0 (166.0)	
	Obese (n = 30)	175.5 (81.0)	
Smoking	Yes (*n* = 28)	186.7 (156.3)	0.046
	No (*n* = 42)	264.5 (157.2)	
Work ability, WAS score	WAS score ≤6 pt. (*n* = 30)	189.5 (117.2)	0.075
(median 7 points) ^2^	WAS score ≥7 pt. (*n* = 39)	252.4 (159.7)	
Handgrip strength ^2^	Within the reference values (*n* = 25)	222.4 (142.0)	0.700
	Under the reference values (*n* = 44)	238.1 (172.6)	
Functional disabilities, HAQ-DI	Mild to moderate (*n* = 49)	255.0 (169.8)	0.108
	Moderate to very severe (*n* = 20)	186.2 (127.90)	
Disease activity; CDAI	Remission (*n* = 30)	233.3 (141.9)	0.219
	Low (*n* = 28)	246.1 (175.6)	
	Moderate (*n* = 8)	139.8 (76.3)	
	High (*n* = 4)	333.6 (259.9)	
CRP ^3^	Within the reference values (*n* = 52)	224.4 (152.8)	0.371
	Over the reference values (*n* = 17)	265.1 (186.6)	
Blood sedimentation ^4^	Within the reference values (*n* = 58)	234.9 (166.5)	0.673
	Over the reference values (*n* = 10)	210.6 (130.9)	
Disease duration;	≤8 years (*n* = 38)	245.8 (166.1)	0.487
(median 8 years)	≥9 years (*n* = 32)	218.8 (154.6)	
Pain last week; ^5^	≤20 points (*n* = 37)	263.4 (179.5)	0.098
(median 20 points)	≥21 points (*n* = 33)	199.8 (130.3)	
Comorbidities ^6^	Moderate comorbidities (*n* = 49)	254.6 (176.7)	0.092
	Severe comorbidities (*n* = 21)	184.0 (100.6)	

WAS = Work Ability Index Single-Item Scale; HAQ-DI = Health Assessment Questionnaire Disability Index; CDAI = Clinical Disease Activity Index (CDAI). ^1^ BMI was categorized as underweight (<18.5 kg/m^2^), normal weight (18.5–24.9 kg/m^2^), overweight (25.0–29.9 kg/m^2^), and obese (≥30 kg/m^2^). ^2^ Sex- and age-specific values of the dominant hand of the European population were used [42]. ^3^ C-reactive protein (CRP) = according to the reference range (0–5 mg/L) of the laboratory. ^4^ According to the reference range (0–40 mm) of the laboratory. ^5^ According to the HAQ-VAS-Pain-Scale. ^6^ Measured with the Charlson Comorbidity Index.

**Table 4:** Multiple linear regression models between physical activity and clinical and sociodemographic variables.

		MVPA (Model 1) ^1^		MVPA (Model 2) ^2^		MVPA (Model 3) ^3^	
		ß (95% CI)	*p*	ß (95% CI)	*p*	ß (95% CI)	*p*
Sex		0.04 (−73.34 to 99.79)	0.761			0.06 (−64.60 to 109.10)	0.610
Age		−0.21 (−7.58 to 0.43)	0.080			−0.12 (−6.78 to 2.78)	0.405
Education level		0.11 (−30.16 to 84.69)	0.345			−0.11 (−92.91 to 38.92)	0.416
Body mass index		−0.12 (−16.36 to −2.71)	0.007	−0.33 (−16.80 to −2.91)	0.006	−0.35 (−17.57 to −3.40)	0.004
Smoking		−0.24 (−154.16 to −1.46)	0.046	−0.23 (−150.70 to 4.26)	0.064	−0.22 (−152.63 to 5.70)	0.068
Work ability; WAS		0.32 (27.99 to 47.13)	0.006	0.28 (−1.56 to 49.03)	0.065	0.16 (−12.91 to 40.46)	0.306
Functional disabilities; HAQ-DI		−0.29 (−141.30 to −14.19)	0.017	−0.23 (−134.33 to 9.13)	0.086	−0.12 (−121.97 to 57.07)	0.471
Disease duration		−0.14 (−6.88 to 1.79)	0.246	−0.04 (−5.44 to 4.12)	0.784	−0.07 (−5.87 to 3.52)	0.618
Pain last week		−0.12 (−2.45 to 0.79)	0.311	−0.06 (−2.11 to 1.25)	0.611	0.07 (−1.97 to 2.06)	0.963
Comorbidities		−0.16 (−49.93 to 9.71)	0.183	−0.13 (−46.04 to 14.16)	0.294	−0.06 (−36.90 to 21.54)	0.601

^1^ Model 1: Univariate linear regression analysis. ^2^ Model 2: Linear regression analysis, with each parameter adjusted for sex, age, and education. ^3^ Model 3: Linear regression analysis, with every variable mutually adjusted for each other. HAQ-DI = Health Assessment Questionnaire Disability Index. Pain was assessed with the HAQ-VAS-Pain-Scale, asking the question, “*How would you rate your pain over the past week on a scale ranging from 1 to 100?*”. WAS = work ability scale.
